# Transcriptomic Profiling of Fruit Development in Black Raspberry *Rubus coreanus*

**DOI:** 10.1155/2018/8084032

**Published:** 2018-04-01

**Authors:** Qing Chen, Xunju Liu, Yueyang Hu, Bo Sun, Yaodong Hu, Xiaorong Wang, Haoru Tang, Yan Wang

**Affiliations:** ^1^College of Horticulture, Sichuan Agricultural University, Chengdu, Sichuan 611130, China; ^2^Science and Technology Management Division, Sichuan Agricultural University, Chengdu, Sichuan 611130, China; ^3^Institute of Pomology and Olericulture, Sichuan Agricultural University, Chengdu, Sichuan 611130, China

## Abstract

The wild *Rubus* species *R*. *coreanus*, which is widely distributed in southwest China, shows great promise as a genetic resource for breeding. One of its outstanding properties is adaptation to high temperature and humidity. To facilitate its use in selection and breeding programs, we assembled de novo 179,738,287 *R*. *coreanus* reads (125 bp in length) generated by RNA sequencing from fruits at three representative developmental stages. We also used the recently released draft genome of *R*. *occidentalis* to perform reference-guided assembly. We inferred a final 95,845-transcript reference for *R*. *coreanus*. Of these genetic resources, 66,597 (69.5%) were annotated. Based on these results, we carried out a comprehensive analysis of differentially expressed genes. Flavonoid biosynthesis, phenylpropanoid biosynthesis, plant hormone signal transduction, and cutin, suberin, and wax biosynthesis pathways were significantly enriched throughout the ripening process. We identified 23 transcripts involved in the flavonoid biosynthesis pathway whose expression perfectly paralleled changes in the metabolites. Additionally, we identified 119 nucleotide-binding site leucine-rich repeat (NBS-LRR) protein-coding genes, involved in pathogen resistance, of which 74 were in the completely conserved domain. These results provide, for the first time, genome-wide genetic information for understanding developmental regulation of *R*. *coreanus* fruits. They have the potential for use in breeding through functional genetic approaches in the near future.

## 1. Introduction

The genus *Rubus* L. comprises 900–1000 species and has a worldwide distribution (excluding Antarctica) with various climatic adaptations [1]. Plants used in fruit production are mainly from two subgenera, *Rubus* and *Idaeobatus*. Blackberries and raspberries are the most commonly cultivated fruits in these two subgenera. They are deemed functional fruits, mainly due to being rich sources of health-promoting antioxidant or “nutraceutical” compounds (i.e., anthocyanins, phenolics, and ellagic acid) in fresh fruits [2] and anticancer properties in fruit extracts [3]. Historically, they have been used in traditional Chinese medicine and are mentioned in the Compendium of Materia Medica (Bencao Gangmu) compiled by Li Shizhen (1518–1593) during the Ming Dynasty. Chinese or Korean black raspberry *R*. *coreanus* Miq., in the subgenus *Idaeobatus*, is named for the dark red (or black) color of its fruits when mature. Earlier investigations found that black raspberry fruits contained higher concentrations of the nutritional ingredients mentioned above than either red raspberry or blackberry [4]. However, Chinese black raspberry is not as popular as the other two species as much less effort is given to its cultivation and there is only a limited choice of cultivars available. After a thorough investigation of the biochemical components in fruit [5], researchers from South Korea provided the first transcriptome analysis of what they believed to be *R*. *coreanus* [6]. Unfortunately, contrary to what is reported in their paper [7], the species they studied was the commercially grown North American black raspberry *R*. *occidentalis*, often confused with *R*. *coreanus*. In 2016, the first draft genome for *R*. *occidentalis*, 243 Mb in size, became publicly available [8]. It is the most useful *Rubus* genetic resource to date.

*R*. *coreanus* has been used as a valuable genetic donor in several *Rubus*-breeding programs [9, 10] because of its outstanding disease resistance and high productivity. *R*. *coreanus* cultivars are also commercialized in South Korea [11]. The lack of a genetic reference for *R*. *coreanus* has become a barrier to application of modern breeding techniques, such as marker-assisted selection and transgenic strategies. Within the past ten years, we have made a comprehensive study of *Rubus* species in China, mostly those endemic to the country, focusing especially on wild species distributed in the south [12]. The relatively high temperature and humidity of the area may have led to the development of unique disease resistance characteristics in these *Rubus* species. Given that viral and fungal diseases have become one of the main threats hindering the development of commercial cultivation of *Rubus* [13], exploring underlying mechanisms for disease resistance and finding new candidate genetic resources could facilitate selection and breeding.

Fruit maturation is a complex process involving genetic regulation closely linked with environmental signaling of palatability. Pigment deposition and the resulting color change, and sugar accumulation, usually coupled with depletion of titratable acid leading to formation of specific sensory traits, are common signs of fruit ripeness [14]. Dissection of the intrinsic genetic changes of fruit maturation could bring new insights into understanding the consequences of application of a particular agronomic practice at a particular time. For example, *Fragaria × ananassa* has traditionally been classified as nonclimacteric because its ripening process is not governed by ethylene. However, global analysis of transcriptome data and the *ethylene response factor* (ERF) gene family has identified involvement of ethylene in ripening of the receptacle at specific tissue/developmental stages [15]. Moreover, unveiling the gene expression atlas of fruit maturation could enable greater understanding of biosynthesis of bioactive compounds, a necessary step toward breeding new varieties for health benefits. Considerable effort has been made in this regard for fruits such as grapes [16], blueberries [17], and tomatoes [18]; in comparison, very little effort has been made for *Rubus* species.

Therefore, as a first step towards understanding gene expression during fruit ripening in *R*. *coreanus*, this study presents results of a comprehensive analysis of transcriptome data from fruits at three representative developmental stages. Both de novo and reference-guided assembly were carried out to maximize the possibility of finding potential transcripts. We also investigated the genes for long noncoding RNAs. Differentially expressed genes, specifically, (1) genes leading to flavonoid biosynthesis and (2) plant nucleotide-binding site leucine-rich repeat (NBS-LRR) genes, which contribute to biotic and abiotic stress responses, were analyzed. It is hoped that exploration of the genetics of *R*. *coreanus* may prove to be a profitable endeavor by providing valuable information for *Rubus* breeding.

## 2. Results and Discussion

### 2.1. Transcriptome Sequencing and Sequence Assembly

Although *Rubus* species are some of the most popular functional fruits in the world, it is only recently that genomic resources have become available for the genus [8]. In Hyun et al.'s study of *R*. *occidentalis* (which they mistook for *R*. *coreanus* [6]) from the perspective of fruit morphology and phenological traits [7], transcriptome analysis involved mRNA isolated only from fruits sampled 20 days after anthesis at an intermediate stage of ripening [6]. This may have underestimated the genetic information for the species. In the present study, 179.74 million 125 bp paired-end raw reads were generated from fruit libraries of three developmental stages. After trimming adapter-related reads and filtering low-quality reads, 65.27 million bases were subjected to error correction. Finally, 174.79 million reads comprising 43.7 gigabases were used to assemble the reference. In total, 78.80 million bases were assembled into 95,845 transcripts, with an N50 length of 1242 bp (Table 1). The generated 125 bp paired-end reads are available at NCBI Sequence Read Archive SRR6001072 to SRR6001077 associated with BioProject PRJNA401210.

To assess the quality of the assembly further, Bowtie (v2.2.9) [19] was employed to align all reads back to the reference. Of the reads, 83.66% could be aligned, with 64.17% aligning concordantly and uniquely to the final version of the reference. In contrast, only 52.97% of the total reads could be aligned to the genome-guided assembly, indicating high divergence between *R*. *coreanus* and *R*. *occidentalis*. This result may partly explain the previously observed phenomenon that although these two species are easy to cross, the F1 progenies are completely sterile [20]. Therefore, it is reasonable that a proportion of our transcripts could not be mapped to the reference. In addition, when evaluated against the complete 1440 plant-specific orthologs in the Benchmarking Universal Single-Copy Orthologs (BUSCO) database [21], the largest proportion of the assembly (95.3%) was complete, with only 27 (1.9%) fragmented and 41 (2.8%) missing orthologs. These results indicate high completeness of our assembly.

**Figure 1:**
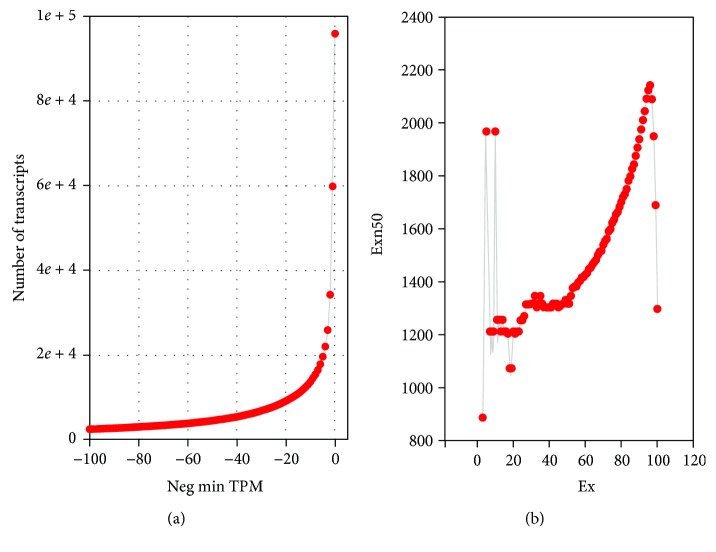
Expression statistics for all transcripts (a) and ExN50 distribution of the assembly (b). Neg min TPM in (a) indicates the negative value of a given minimum expression level as transcripts per million (TPM) reads. Ex indicates that *x*% of the assembled transcript nucleotides can be found in contigs that are at least of ExN50 length.

Taking expression values into consideration, we plotted the transcripts per million (TPM) distribution patterns of all transcripts (Figure 1(a)). Predominant portions of the transcripts were at low abundance. If using three TPM as a threshold, each fraction of 39,039 transcripts could be viewed as from one genuine gene. This number is within the range of gene numbers from the *R*. *occidentalis* genome project [8]. Taking this read coverage information before abundance filtering, the N50 value for the top Ex% transcripts was calculated (Figure 1(b)). The maximum N50 value (2142 bp) was reached when taking 96% of the upper expressed gene products.

### 2.2. Functional Annotations for the *R*. *coreanus* Transcriptome

Annocript pipeline [22] was employed to annotate transcripts and coding peptides. Searches for homologous counterparts in the manually annotated, nonredundant protein sequence database Swiss-Prot (SP) and the subset UniProt Reference Clusters Uniref90 (UF90) database by the blastx algorithm resulted in 47,090 (49.99%) of the raw transcripts generating hits in SP and 66,520 (69.40%) of the transcripts with homologs in UF90. More specifically, 15.60% of the SP hits and 25.43% of the UF90 hits were covered over 80% by the enquiry sequences. In the case of gene ontology (GO) assignment, 51,520 (53.758%) transcripts could be classified into Biological Process (28,547), Cellular Component (31,728), or Molecular Function (41,971) categories. Searches in the Kyoto Encyclopedia of Genes and Genomes (KEGG) Orthology (KO) database against related plants resulted in KO identifiers of 42,769 of the transcripts being assigned to the corresponding pathways. Through PORTRAIT noncoding potential evaluation using a new support vector machine-based algorithm [23], 2178 long noncoding RNA- (lncRNA-) coding genes were also discovered. Taken together, these results suggest that the de novo assembled reference covered a wide range of *Rubus* genetic information, which provides a valuable resource for facilitating the discovery of novel genes involved in specific physiological and developmental processes.

### 2.3. Analysis of Differential Gene Expression across the Three Developmental Stages of *R*. *coreanus* Fruit

We mapped all reads from each fruit developmental stage and estimated transcript counts against the reference using the RSEM method [24]. Transcripts with less than three TPM across the three stages were filtered in the subsequent differentially expressed gene (DEG) assay based on the above analysis. Three different expression analysis packages were used for DEG detection: (1) DESeq2 [25], which uses a Wald test; (2) edgeR [26], which uses a likelihood ratio test; and (3) limma-voom [27], which uses a moderated *t*-test, to compare expression differences between fruit stages. In the consensus results, 211 transcripts were expressed differentially in red fruits compared to green fruits. Among these genes, 49 were downregulated and 162 were upregulated. Between black (mature) and red fruit stages, 1141 genes were upregulated and 1423 downregulated. Variation in expression was observed in 2363 genes between black and green fruits. Although the strict criteria we used in this analysis may overlook other gene products, they can be viewed as generating the most reliable DEGs.

**Figure 2:**
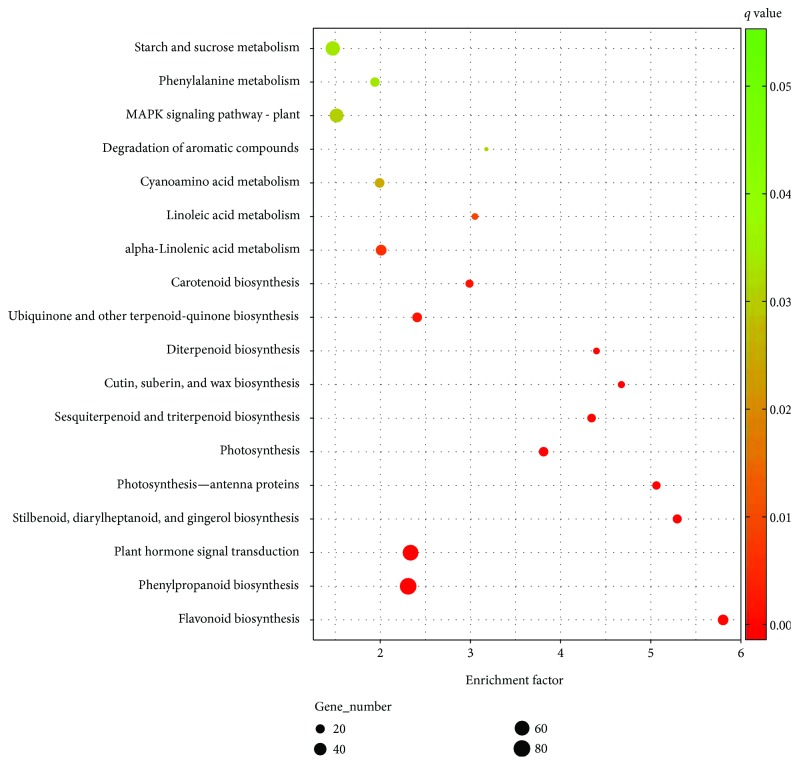
Pathway enrichment of differentially expressed genes between green and red *Rubus coreanus* fruits.

**Figure 3 fig3:**
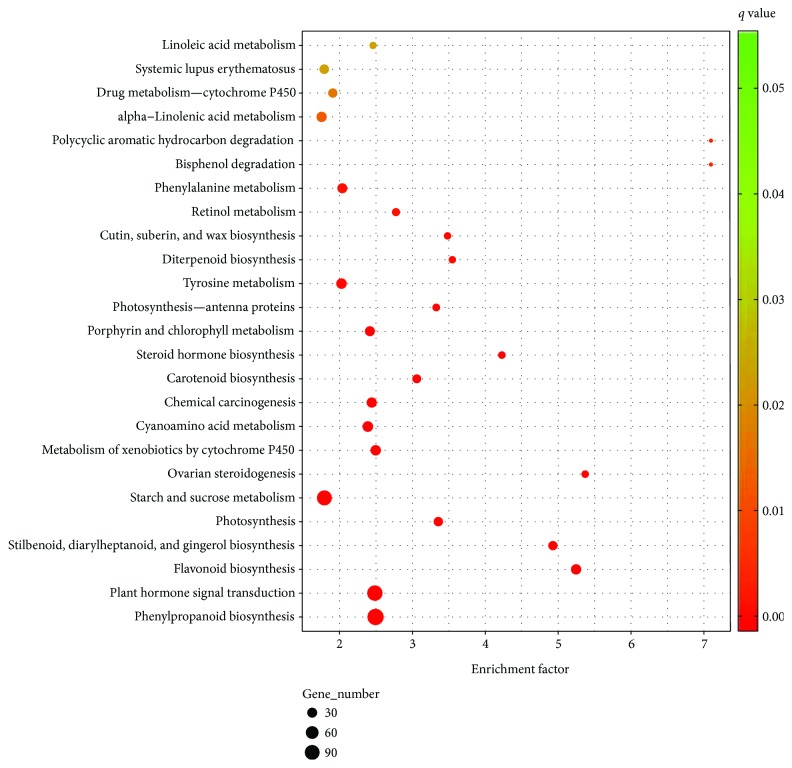
Pathway enrichment of differentially expressed genes between red and black *Rubus coreanus* fruits.

**Figure 4:**
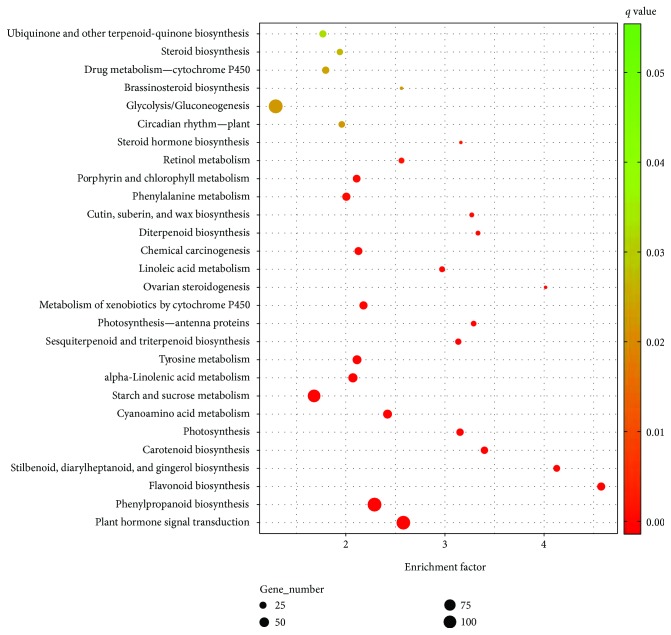
Pathway enrichment of differentially expressed genes between green and black *Rubus coreanus* fruits.

GO and KO enrichment analyses were carried out to consider more closely these differentially expressing genes. When testing for GO terms detected from differentially expressed genes in green versus red, red versus black, and green versus black fruits, no significantly enriched genes were found by GOEAST (http://omicslab.genetics.ac.cn/GOEAST/tools.php). However, several biological pathways were found to be significantly perturbed. Sixteen pathways were enriched across the whole fruit developmental process, including those of genes involved in flavonoid biosynthesis, phenylpropanoid biosynthesis, plant hormone signal transduction, and cutin, suberin, and wax biosynthesis, among others (Figures 2 [Fig fig3]–4). In addition to these commonly impacted pathways, alterations in “degradation of aromatic compounds” and “MAPK signaling pathway - plant” were detected specifically in the early stages (change from small green to red fruit). In contrast, “bisphenol degradation” and “polycyclic aromatic hydrocarbon degradation” pathways were affected in the later stages (change from red to fully ripe black fruit). Fruit ripening is a process of highly coordinated and genetically programed physiological, biochemical, and organoleptic changes in the reproductive organs. In *Rubus* fruits, predominant changes in ripening include (1) depolymerization of carbohydrates, specifically, degradation of starches into sucrose and then into glucose and fructose; (2) decrease in organic acids, including amino acids; (3) production of volatile compounds, such as alcohols and aldehydes; and (4) accumulation of anthocyanins but depletion of cinnamic, ferulic, protocatechuic, and vanillic acids and epicatechin [5]. These changes may be evident in the metabolic pathway profiling in our study. The starch and sucrose metabolism pathway, significantly enriched in the two early stages of fruit ripening, adding to the degradation of aromatic compounds, can lead to the formation of special flavor and aroma of ripening *Rubus* fruits. A mixture of compounds, including ketones, alcohols, esters, and mainly terpenoids, constitutes the volatile flavor of most, if not all, fruits [28]. Some *Rubus* species have a special aroma to their fruit, but some do not [29]. Degradation of aromatic compounds could have a partial impact on these aroma volatiles. Another obvious sign of maturation of soft fruits is the decrease in firmness, which is the result of degradation of cell wall components and/or loss of integrity of the cell cuticular/wax layer [30, 31]. In strawberries, cell wall disassembly is characterized by solubilization of pectins, slight depolymerization of covalently bound pectins, and loss of galactose and arabinose, as well as a reduction in hemicellulose content [32]. Pectin content of mature fruit reduced dramatically in two raspberry cultivars, “Glen Clova” and “Glen Prosen” [33]. Further examination of *R*. *idaeus* cell wall fraction indicated that fruit ripening was associated with increased solubilization of pectin first and then depolymerization at the last stage [34]. In support of this, DEGs for cutin, suberin, and wax biosynthesis were found to be significantly enriched across the three fruit-ripening stages in our study. Only two DEGs (omega-hydroxypalmitate O-feruloyl transferase and peroxygenase) were common to all three stages. Progressive modulation of these particular genes may be the molecular basis of programing of the fruit-softening process.

### 2.4. Flavonoid Biosynthesis Genes and Their Expression

**Figure 5:**
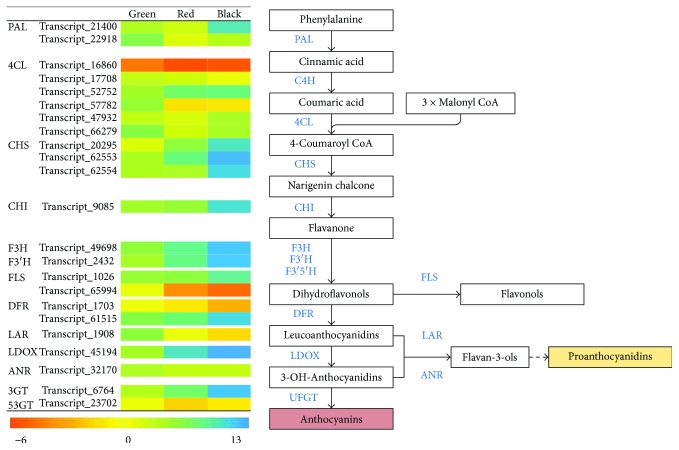
Flavonoid-synthesis-associated transcripts and their expression patterns during fruit ripening. Expression values are presented as log2-transformed trimmed mean of *M* value (TMM) derived from edgeR analysis. PAL: phenylalanine ammonia-lyase; 4CL: 4-coumarate:CoA ligase; CHS: chalcone synthase; CHI: chalcone flavanone isomerase; F3H: flavanone 3-hydroxylase; F3′H: flavonoid 3′-hydroxylase; F3′5′H: flavonoid 3′,5′-hydroxylase; FLS: flavonol synthase; DFR: dihydroflavonol 4-reductase; LDOX: leucoanthocyanidin dioxygenase; LAR: leucoanthocyanidin reductase; ANR: anthocyanidin reductase; 3GT: anthocyanin 3-O-glucosyltransferase; 53GT: anthocyanin 3,5-O-glucosyltransferase.

Anthocyanin, the most important metabolite in flavonoid production, is an essential nutritional component in raspberry fruits and their products [35, 36]. In *R*. *coreanus*, cyanidin-3-glucoside, cyanidin-3-rutinoside, and pelargonidin-3-glucoside have been recognized as the major anthocyanins [35]. Anthocyanins are first detected in green-red fruit but increase at the greatest rate to the highest amount in the last developmental stage [5]. The same trend has been observed for flavonols such as quercetin-glucuronide and quercetin-3-O-rutinoside. In contrast, flavanols and proanthocyanidins are accumulated at the very beginning of fruit set [5]. All these compounds are final products typical of the flavonoid biosynthesis, anthocyanin, and flavonol synthesis pathways. In our study, both flavonoid and anthocyanin pathways were significantly enriched during fruit development. This is in accordance with findings for other fruits such as grapes [16] and bayberries [37]. Confirming our prediction from the KEGG pathway enrichment above, we were able to manually identify 23 transcripts involved in the flavonoid biosynthesis pathway leading to flavonols, anthocyanins, or proanthocyanidins. The expression of most of these transcripts perfectly parallels the changes in the metabolites (Figure 5). Among these genes, five have alternative transcripts/multigene members including two *phenylalanine ammonia-lyases* (PAL), six *4-coumarate:CoA ligases* (4CL), three *chalcone synthases* (CHS), two *flavonol synthases* (FLS), and two *dihydroflavonol 4-reductases* (DFR). Major players among the transcripts from the same gene/multigene could be identified from expression patterns. For example, among the six *4CL* transcripts, transcript_52752 may be the key actor, whose abundance increased highly in parallel with fruit maturation. In comparison, although their roles could not be ruled out, most other transcripts of *4CL* exhibited very low levels of expression throughout the three fruit developmental stages. Functional diversification could be deduced from the results if multigene copies existed. Examples include *PAL* (transcript_21400 and transcript_22918) and *DFR* (transcript_1703 and transcript_61515). One member of *PAL* or *DFR* had a completely opposite expression mode compared to the other (Figure 5). In strawberries, the two copies of *DFR* have different substrate affinities, exerted at different stages for producing different types of anthocyanin [38]. Therefore, the function of the *DFR*s isolated in *R*. *coreanus* needs further investigation. Also noteworthy is the absence of the *F3′5′H* gene in the transcriptome, which implies that the synthesis of delphinidin-derived anthocyanins is blocked in *R*. *coreanus* fruits.

### 2.5. Identification and Abundance Estimation of NBS-LRR-Encoding Genes

**Figure 6:**
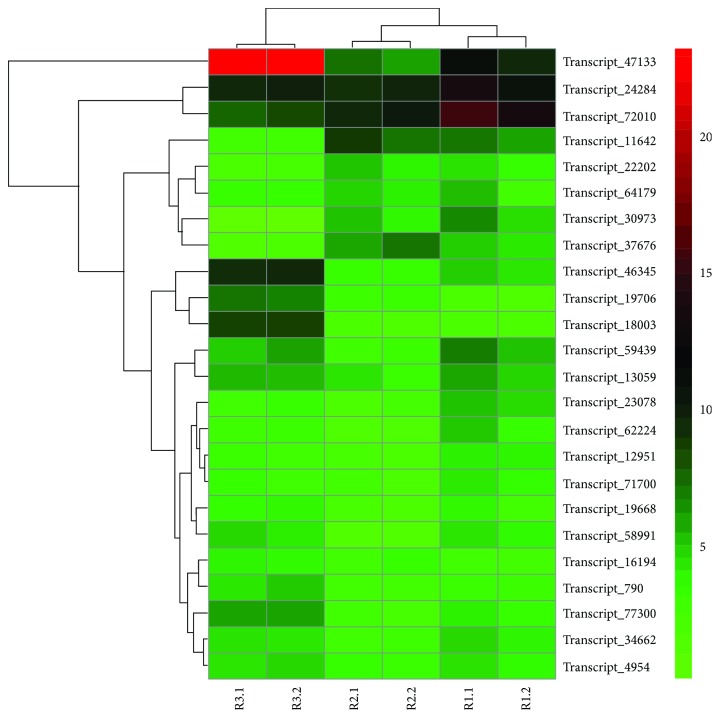
Heat map of the top 24 NBS-LRR genes expressed in *Rubus coreanus*. Normalized expression values are presented as trimmed mean of *M* value (TMM) derived from the edgeR package.

Fungal and viral diseases are two worldwide threats to commercial cultivation of *Rubus*. Given the requirement for reducing pesticide use, cultivars with robust disease resistance become increasingly important. Fungal pathogens attack every part of *Rubus*, including the roots, canes, leaves, and fruits. Several fungal diseases can cause pre- or postharvest fruit rot in raspberries, leading to a short shelf life and limited sales of fresh fruit to distant markets. Gray mold (*Botrytis cinerea* Pers.:Fr.) is the most serious pathogen of fruit. Variation in susceptibility to it has been observed in fruits from different raspberry cultivars [39]. It is well documented that *R*. *coreanus* derivatives have strong resistance to cane diseases caused by *Elsinoë veneta*, *Didymella applanata*, and *B*. *cinerea* [40]. *R*. *coreanus* has also been recommended as a resource for promoting fruit rot resistance [10, 41, 42]. Proteins that contain a nucleotide-binding site and leucine-rich repeat (NBS-LRR) domains consist of the largest class of known plant resistance (R) gene products, conferring resistance to a diverse spectrum of pathogens [43, 44]. Recent advances have revealed that NBS-LRR R proteins are able to inhibit *B*. *cinerea* development [45]. In the family Rosaceae, NBS-LRR-coding genes form a large proportion of the genome, from 1.05% in strawberries to 1.52% in peaches [46]. However, except of a few studies, the availability of R gene resources in *Rubus* species is limited. Samuelian et al. [47] characterized 75 LRR genes from *R*. *idaeus* using degenerate primers designed from other Rosaceae species. Afanador-Kafuri and colleagues [48] obtained 47 LRR proteins using a similar strategy from Colombian *Rubus* genotypes. In a further exploration of our transcriptome data, initial screening via hmmsearch of the new reference uncovered 411 candidate NBS-encoding transcripts. Thereafter, through domain hunting, 119 NBS-LRR-domain-coding transcripts were identified, among which 74 had hits in the completely conserved NBS domain (Supplementary Table 1). We believe our resources greatly enrich the genetic information for *Rubus* breeding. Most of these plant resistance protein-coding genes have low abundance (less than five) estimated as trimmed mean of *M* values (TMM). This appears reasonable because a very high expression of R proteins could bring lethal effects to plant cells [49]. Twenty-four NBS transcripts are presented in Figure 6. Two of the transcripts show relatively high expression values (transcript_24284 and transcript_72010) in fruits at almost all three stages. Transcript_47133, with the highest abundance, functions mainly in the last stage, when fruits are fully ripe and are more vulnerable to pathogen attack. Closer examination of this resistance gene found that it is in the class of NBS-LRR (NL) proteins lacking additional N-terminal domains. Its closest ortholog in *R*. *occidentalis* is the gene *Bras_G19818*, which shares 62.58% sequence identity. Some of the *NBS-LRR* genes have tissue-specific expression properties [50] and can even confer different resistance reactions with different alleles from the same gene [51]. These three highly expressed or stage-specific factors could be interesting candidates for more detailed investigation in the future.

### 2.6. Cloning and Real-Time Quantitative PCR (RT-qPCR) Validation of Representative Genetic Information

**Figure 7:**
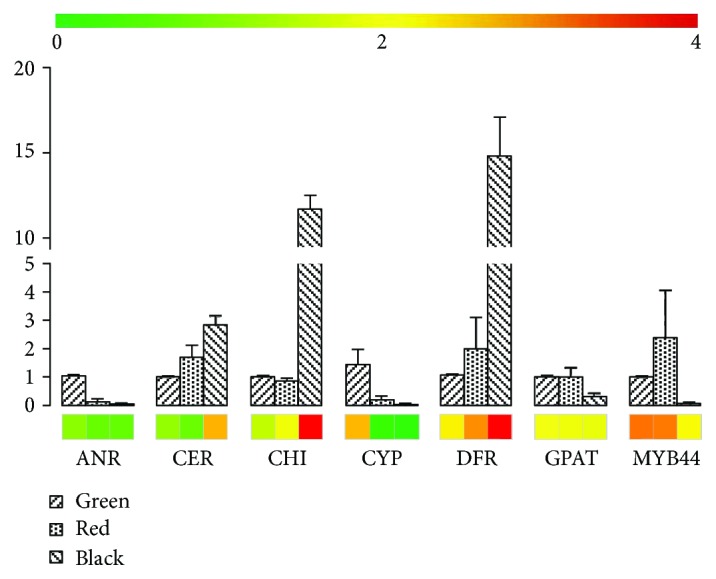
Similarities in expression patterns of seven genes between RNA-seq (heat map) and RT-qPCR (bar plot). ANR: anthocyanidin reductase; CER: ECERIFERUM; CHI: chalcone flavanone isomerase; CYP: cytochrome P450; DFR: dihydroflavonol 4-reductase, (transcript_61515 was chosen for DFR); GPAT: glycerol-3-phosphate acyltransferases. MYB44 is the transcript most resembling AtMYB44 in *Arabidopsis thaliana*.

Seven representative genes (*ANR*, *CER*, *CHI*, *CYP86B1*, *DFR*, *GPAT*, and *MYB44*), which encode key enzymes or regulators involved in anthocyanin/proanthocyanidin biosynthesis, amino acid metabolism, or plant cell wall wax formation, were successfully cloned and validated by sequencing. All these gene sequences corresponding to the full length of coding sequence with various lengths of 5′ or 3′ untranslated region (UTR) were identical to those deduced from the RNA-seq results. Similar expression patterns between RNA-seq and RT-qPCR were also observed (Figure 7), thus further validating the RNA-seq expression data.

## 3. Conclusions

This is the first transcriptomic profile, through RNA-seq investigation of sequence and transcript abundance, for *R*. *coreanus*. The transcriptomic analysis provides, for the first time, a 95,845-transcript reference for the species. Of these genetic resources, 69.5% were annotated. Differentially expressed genes in fruit developmental stages were mainly involved in flavonoid biosynthesis, plant cell wall formation, and aroma compound degradation. We identified 23 transcripts involved in the flavonoid biosynthesis pathway whose expression perfectly paralleled changes in the metabolites. Additionally, we identified 119 nucleotide-binding site leucine-rich repeat (NBS-LRR) protein-coding genes, involved in pathogen resistance, of which 74 were in the completely conserved domain. We believe that our study provides useful genetic information for *Rubus* breeding.

## 4. Materials and Methods

### 4.1. Sample Collection and RNA Preparation

Fruits of *R*. *coreanus* (2*n* = 2*x* = 14) [12] were collected from the wild at Ya'an city, Sichuan province (29°58′24.5″N, 103°00′18.7″E). Fruit set occurs in April and fruits mature in mid-June in this area. Fruits of three representative stages of ripening (green, red, and mature black) were harvested in 2015. For each stage, a total of about 30 fruits from no more than three canes were collected in order to decrease background variation. They were frozen immediately in liquid nitrogen on collection and stored at −80°C until use. Two biological replicated samples were collected for each stage due to the limited yield of fruits.

Total RNA was isolated by using a modified cetyltrimethylammonium bromide method [52]. Genomic DNA was eliminated by using RNase-free DNase I (TaKaRa, Dalian, China). After monitoring RNA integrity and purity on 1% agarose gels and NanoPhotometer spectrophotometer (Implen, CA, USA), the Agilent 2100 Bioanalyzer system (Agilent Technologies, CA, USA), supplemented with RNA 6000 Nano Kit, was used to confirm the results. RNA concentration was measured using Qubit RNA Assay Kit in Qubit 2.0 Fluorometer (Life Technologies, CA, USA).

### 4.2. cDNA Synthesis, Library Construction, and Sequencing

As input material, 3 *μ*g of RNA per sample was used. Sequencing libraries were generated using NEBNext Ultra Directional RNA Library Prep Kit for Illumina (NEB, USA) according to the manufacturer's instructions. Briefly, mRNA was purified from total RNA using poly(T) oligo-attached magnetic beads. Fragmentation was carried out using divalent cations under elevated temperature in NEBNext First Strand Synthesis reaction buffer (5x). First-strand cDNA was synthesized with random hexamer primer and M-MuLV Reverse Transcriptase (RNase H). Second-strand cDNA was synthesized by DNA polymerase I and RNase H. Remaining overhangs were blunted via exonuclease/polymerase activities. After adenylation of 3′ ends of DNA fragments, NEBNext Adaptors with a hairpin loop structure were ligated. AMPure XP system (Beckman Coulter, Beverly, USA) was used to purify cDNA fragments selectively at the correct size. Then, 3 *μ*l of USER Enzyme (NEB, USA) was used with size-selected, adaptor-ligated cDNA at 37°C for 15 min followed by 5 min at 95°C. PCR was then performed with Phusion High-Fidelity DNA Polymerase, universal PCR primers, and index primers. Finally, the products were purified (AMPure XP system) and library quality was assessed on the Agilent 2100 Bioanalyzer system. Clustering and sequencing were carried out with the prepared libraries by Novogene (Beijing, China) using the Illumina HiSeq 2500 platform.

### 4.3. Transcriptome Assembly, Annotation, and Differential Expression and Enrichment Analysis

The raw reads were cleaned by removing adapter sequences and ambiguous reads (with “*N*” > 10%). Low-quality bases were trimmed, and reads that were too short were filtered through Trimmomatic (LEADING:3 TRAILING:3 SLIDINGWINDOW:4:15 MINLEN:50) [53], as were the corresponding read pairs. After trimming/filtering low-quality reads, SEECER (v0.1.3) [54] was used for error correction. All downstream analyses were based on high-quality clean data.

To facilitate the use of the recently published North American black raspberry genome information, we adopted the strategies of genome-guided transcript expression analysis by using the protocol of Trapnell et al. [55]. All reads were first mapped to the *R*. *occidentalis* genome (v1.0) with TopHat2 (allowing two bases of mispairing and multiple hits ≤ 20) and then assembled by using the Cufflinks suite with default parameters.

To evaluate divergence between *R*. *coreanus* and *R*. *occidentalis*, we also carried out de novo assembly of transcripts. Trinity (v2.2.0) [56] was used with default parameters except that the minimum contig length was set to 200 bp, reads were first normalized with maximum coverage 50 before putting in the assembly pipeline, and k-mer coverage was set to a minimum level of two. Redundancy in the de novo transcriptome was minimized with CD-HIT-EST (v4.6.4) [57] using an identity cutoff at 0.99. EvidentialGene tr2aacds pipeline [58] was used to combine both genome-guided and de novo assemblies. Nonredundant transcripts were also obtained. To evaluate the quality of the reference, all assemblies were searched against BUSCO for plants [21].

All reads in each sample were mapped back to the transcriptome using Bowtie 2 [19] (default parameters used, but end to end, allowing two bases of mispairing and multiple hits ≤ 20) and then used to estimate expression values for each transcript by RSEM [24]. Given that many of the very lowly expressed transcripts could be questionable due to our very high deep-sequencing coverage (exceeding 100x), we filtered the transcripts by setting transcripts per million (TPM) lower than three before conducting differential gene expression analysis.

Gene annotations were carried out according to the Annocript (v1.1.3) pipeline [21] using all assembled transcripts. We performed similarity searches through blastx against UniRef90 and Swiss-Prot (v201706, word_size = 4; e-value = 0.00001), rpsblast against the Conserved Domain Database (CDD) profiles (ftp://ftp.ncbi.nih.gov/pub/mmdb/cdd/little_endian/Cdd_LE.tar.gz, e-value = 0.00001, num_descriptions = 20, and num_alignments = 20), and blastn against Rfam and rRNAs (e-value = 0.00001, num_descriptions = 1, num_alignments = 1, and num_threads = 4). For each sequence, the best hit, if any, was chosen. For gene ontology (GO) functional classification, Enzyme Commission IDs were associated to the corresponding matches. KEGG Orthology (KO) assistant pathway assignment was implemented via KOBAS 3.0 [59] using the default parameters. The dna2pep tool implemented in the Annocript package [21] was used to identify the longest open reading frame (ORF) of each transcript. PORTRAIT software [23], which was developed for detecting noncoding RNA from poorly characterized species, was used to identify the noncoding potential of each query sequence by using a new support vector machine-based algorithm.

To investigate differential expression (DE) of transcripts, we used DESeq2 [25], which uses a Wald test; edgeR [26], which uses a likelihood ratio test; and limma-voom [27], which uses a moderated *t*-test to conduct pairwise comparison of the three fruit-ripening stages. Each of the comparisons was based on different statistical models. Differentially expressed genes were selected using log2FC ≥ 1 or logFC ≤ −1 and FDR (false discovery rate) < 0.01 in the three methods. Consensus DE results were obtained by comparing the outcomes of the three methods, which were used to present the most reliable differentially expressed transcripts. These transcripts associated with their corresponding GO or KO annotations were tested against the whole transcriptome as background gene sets for enrichment analysis. GO categories were checked using GOEAST (http://omicslab.genetics.ac.cn/GOEAST/tools.php) with an FDR (Benjamini–Yekutieli method) value of ≤0.05 as the cutoff to identify enriched terms by the hypergeometric test. Transcripts with a KO number were also tested by hypergeometric statistics to find significantly overperturbed pathways through a Perl in-house script.

### 4.4. Expression Patterns of Genes Involved in Flavonoid Synthesis

From the gene differential expression analysis, flavonoid biosynthesis pathway genes appeared to be extremely perturbed in both green versus red fruits and red versus black fruits. We identified all genes involved in the pathway from the assembled reference and manually curated by blasting against the nonredundant protein database at the National Center for Biotechnology Information site, coupling the annotation from Annocript described above. Afterwards, expression patterns of these genes were presented as heat maps after log2 transformation of the among-sample normalized count values by using edgeR.

### 4.5. NBS-Encoding Gene Retrieval and Expression Analysis

Based on the Annocript-deduced peptide collection, we identified potential NBS-encoding genes using the procedures described by Arya et al. [60]. Specifically, the hidden Markov model (HMM) profile for the NBS domain (http://pfam.xfam.org/family/PF00931) was used to search against the complete set of the predicted *R*. *coreanus* proteins using hmmsearch in HMMER (v3.1b) [61] with e-value < 0.00001. All the protein sequences identified were further subjected to CDD detection (https://www.ncbi.nlm.nih.gov/Structure/bwrpsb/bwrpsb.cgi) using 0.01 as a cutoff value to confirm the presence of NBS domains. Expression pattern analysis was carried out the same way as for flavonoid genes.

### 4.6. Cloning and RT-qPCR for Validation of Gene Expression Patterns

To verify the validity of the genetic information obtained, we selected seven representative genes (*ANR*, *CER1*, *CHI*, *CYP*, *DFR*, *GPAT*, and *MYB44*), which encode key enzymes involved in anthocyanin/proanthocyanidin biosynthesis, amino acid metabolism, plant cell wall wax formation, or stress response regulators. The deduced full coding sequences were cloned experimentally, and their expression values were determined using RT-qPCR. All the candidate sequences were amplified in a 20 *μ*l reaction mixture, containing 10 ng first-strand cDNA, 10 pmol each primer (Supplementary Table 2), and 10 *μ*l 2x PrimeSTAR HS premix (TaKaRa, Dalian, China). Following one cycle of 20 seconds at 98°C, 35 PCR cycles of 10 s at 98°C, 10 s at 60°C, and 90 s at 72°C were performed in the thermal cycler PTC-200 (Bio-Rad, Hercules, CA). Amplified products were purified using E.Z.N.A. Gel Extraction Kit (Omega, GA, USA). The enriched PCR product was cloned into pEASY-Blunt vectors (TransGen, Beijing, China) and transformed into JM109 competent *Escherichia coli* cells. Finally, positive clones were sequenced using the BigDye Terminator Cycle Sequencing Kit on an ABI PRISM 3730 automated DNA sequencer (Applied Biosystems, Foster City, CA, USA). For quantitative PCR, 10 *μ*l reaction mixture is composed of 5 *μ*l 2x SYBR Green mixture (TaKaRa, Dalian, China), 1 *μ*l diluted cDNA, and 1 *μ*l specific forward and reverse primer for each gene (Supplementary Table 1). The reaction was conducted on a CFX96 Real-Time PCR Detection System (Bio-Rad, US). Expression values were expressed as 2^−ΔΔCT^ using *beta-actin* [52] as an internal control.

## Figures and Tables

**Table 1:** Overview of the assembly.

	*Rubus occidentalis* genome guided	De novo	Final reference
Total number of transcripts	47,239	296,591	95,845
Total nucleotides	80,446,066	214,031,901	78,800,996
Average length (bp)	1703	813	822
Minimum length (bp)	102	201	102
Maximum length (bp)	21,369	14,054	17,356
N50 (bp)	2496	1603	1242

## Supplementary Materials

Supplementary Materials Supplementary Table 1: domain identification and completeness of NBS-LRR proteins in *Rubus coreanus* fruits via CDD scan. Supplementary Table 2: primers used in the amplification and RT-qPCR analysis.
