# Supplementary material for: Transcriptomic Profiling of Fruit Development in Black Raspberry Rubus coreanus
Source: Int J Genomics. 2018 Apr 1;2018:8084032. doi: 10.1155/2018/8084032 (PMC5901860; doi:10.1155/2018/8084032)
Supplement: Supplementary Materials — Supplementary Table 1: domain identification and completeness of NBS-LRR proteins in Rubus coreanus fruits via CDD scan. Supplementary Table 2: primers used in the amplification and RT-qPCR analysis. [file 8084032.f1.docx]

Supplementary Table 1 Domain identification and completeness of NBS-LRR proteins in *Rubus coreanus* fruits via CDD scan (https://www.ncbi.nlm.nih.gov/Structure/cdd/cdd_help.shtml)

| Query | Hit type | PSSM-ID | From | To | E-Value | Bitscore | Accession | Short name | Incomplete |
| --- | --- | --- | --- | --- | --- | --- | --- | --- | --- |
| Transcript_10381 | specific | 227223 | 1138 | 1389 | 4.26E-09 | 60.367 | COG4886 | LRR | C |
| Transcript_10381 | specific | 227223 | 600 | 807 | 6.09E-07 | 53.4334 | COG4886 | LRR | NC |
| Transcript_10381 | non-specific | 227223 | 699 | 843 | 0.000841588 | 43.4182 | COG4886 | LRR | C |
| Transcript_10381 | superfamily | 307194 | 194 | 460 | 1.35E-22 | 99.7688 | cl26397 | NB-ARC superfamily | - |
| Transcript_10568 | specific | 227223 | 763 | 999 | 8.78E-07 | 52.2778 | COG4886 | LRR | NC |
| Transcript_10568 | non-specific | 227223 | 752 | 946 | 0.000119099 | 45.7294 | COG4886 | LRR | NC |
| Transcript_10568 | superfamily | 307194 | 360 | 651 | 2.86E-75 | 250.382 | cl26397 | NB-ARC superfamily | - |
| Transcript_10914 | specific | 227223 | 456 | 672 | 7.38E-07 | 53.0482 | COG4886 | LRR | C |
| Transcript_10914 | non-specific | 227223 | 396 | 654 | 1.20E-06 | 52.2778 | COG4886 | LRR | - |
| Transcript_10914 | non-specific | 227223 | 330 | 473 | 3.13E-05 | 47.6554 | COG4886 | LRR | NC |
| Transcript_10914 | non-specific | 227223 | 399 | 630 | 0.000132934 | 45.7294 | COG4886 | LRR | N |
| Transcript_10914 | superfamily | 307194 | 32 | 193 | 6.38E-16 | 79.7385 | cl26397 | NB-ARC superfamily | C |
| Transcript_11024 | specific | 227223 | 183 | 415 | 4.37E-05 | 45.7294 | COG4886 | LRR | N |
| Transcript_11024 | superfamily | 307194 | 33 | 79 | 6.08E-08 | 53.9301 | cl26397 | NB-ARC superfamily | N |
| Transcript_11179 | specific | 227223 | 558 | 701 | 3.04E-07 | 53.0482 | COG4886 | LRR | N |
| Transcript_11179 | non-specific | 227223 | 564 | 704 | 5.09E-07 | 52.2778 | COG4886 | LRR | NC |
| Transcript_11179 | non-specific | 227223 | 536 | 647 | 0.000884604 | 42.2626 | COG4886 | LRR | N |
| Transcript_11179 | superfamily | 307194 | 165 | 432 | 1.75E-45 | 164.097 | cl26397 | NB-ARC superfamily | - |
| Transcript_11642 | specific | 227223 | 580 | 752 | 7.39E-05 | 46.4998 | COG4886 | LRR | NC |
| Transcript_11642 | superfamily | 307194 | 226 | 454 | 2.89E-19 | 89.3684 | cl26397 | NB-ARC superfamily | - |
| Transcript_11948 | specific | 227223 | 1352 | 1652 | 9.90E-07 | 53.0482 | COG4886 | LRR | N |
| Transcript_11948 | specific | 227223 | 565 | 706 | 8.11E-06 | 49.9666 | COG4886 | LRR | NC |
| Transcript_11948 | non-specific | 227223 | 558 | 802 | 3.80E-05 | 48.0406 | COG4886 | LRR | NC |
| Transcript_11948 | non-specific | 227223 | 523 | 667 | 6.06E-05 | 47.2702 | COG4886 | LRR | N |
| Transcript_11948 | non-specific | 227223 | 520 | 644 | 0.000201748 | 45.7294 | COG4886 | LRR | NC |
| Transcript_11948 | superfamily | 307194 | 167 | 460 | 1.03E-49 | 178.35 | cl26397 | NB-ARC superfamily | - |
| Transcript_11948 | superfamily | 307194 | 1063 | 1252 | 2.11E-43 | 160.245 | cl26397 | NB-ARC superfamily | N |
| Transcript_12689 | specific | 227223 | 515 | 666 | 4.73E-10 | 62.293 | COG4886 | LRR | NC |
| Transcript_12689 | superfamily | 307194 | 161 | 436 | 8.66E-93 | 294.295 | cl26397 | NB-ARC superfamily | - |
| Transcript_12947 | specific | 227223 | 496 | 668 | 4.35E-09 | 59.5966 | COG4886 | LRR | NC |
| Transcript_12947 | superfamily | 307194 | 174 | 450 | 7.20E-57 | 197.995 | cl26397 | NB-ARC superfamily | - |
| Transcript_12951 | specific | 227223 | 546 | 640 | 4.33E-05 | 47.2702 | COG4886 | LRR | NC |
| Transcript_12951 | non-specific | 227223 | 553 | 640 | 0.00131094 | 42.6478 | COG4886 | LRR | NC |
| Transcript_12951 | non-specific | 227223 | 553 | 676 | 0.00324763 | 41.107 | COG4886 | LRR | NC |
| Transcript_12951 | superfamily | 307194 | 167 | 448 | 9.13E-55 | 192.602 | cl26397 | NB-ARC superfamily | - |
| Transcript_13055 | specific | 227223 | 664 | 921 | 3.17E-07 | 54.2038 | COG4886 | LRR | N |
| Transcript_13055 | non-specific | 227223 | 630 | 825 | 9.34E-07 | 52.663 | COG4886 | LRR | NC |
| Transcript_13055 | non-specific | 227223 | 720 | 959 | 0.00102192 | 43.033 | COG4886 | LRR | C |
| Transcript_13055 | non-specific | 227223 | 764 | 948 | 0.00111414 | 43.033 | COG4886 | LRR | C |
| Transcript_13055 | superfamily | 307194 | 241 | 452 | 1.40E-15 | 78.5829 | cl26397 | NB-ARC superfamily | - |
| Transcript_13059 | specific | 227223 | 4 | 204 | 2.78E-09 | 59.9818 | COG4886 | LRR | NC |
| Transcript_13059 | superfamily | 307194 | 432 | 554 | 3.53E-14 | 73.9605 | cl26397 | NB-ARC superfamily | N |
| Transcript_13182 | specific | 227223 | 573 | 766 | 5.99E-08 | 55.7446 | COG4886 | LRR | NC |
| Transcript_13182 | non-specific | 227223 | 560 | 882 | 9.69E-08 | 54.9742 | COG4886 | LRR | N |
| Transcript_13182 | non-specific | 227223 | 571 | 695 | 0.00655099 | 39.5662 | COG4886 | LRR | N |
| Transcript_13182 | superfamily | 307194 | 174 | 464 | 1.98E-88 | 283.124 | cl26397 | NB-ARC superfamily | - |
| Transcript_13623 | specific | 227223 | 464 | 599 | 6.95E-09 | 58.441 | COG4886 | LRR | NC |
| Transcript_13623 | non-specific | 227223 | 479 | 655 | 1.36E-05 | 48.0406 | COG4886 | LRR | N |
| Transcript_13623 | superfamily | 307194 | 316 | 348 | 0.000468245 | 42.7593 | cl26397 | NB-ARC superfamily | N |
| Transcript_13624 | specific | 227223 | 515 | 609 | 1.96E-07 | 54.2038 | COG4886 | LRR | NC |
| Transcript_13624 | non-specific | 227223 | 515 | 611 | 1.16E-06 | 51.5074 | COG4886 | LRR | NC |
| Transcript_13624 | superfamily | 307194 | 109 | 394 | 3.78E-42 | 155.623 | cl26397 | NB-ARC superfamily | - |
| Transcript_13950 | specific | 227223 | 518 | 817 | 1.24E-09 | 60.7522 | COG4886 | LRR | C |
| Transcript_13950 | non-specific | 227223 | 682 | 821 | 0.00409297 | 40.3366 | COG4886 | LRR | C |
| Transcript_13950 | superfamily | 307194 | 225 | 444 | 3.94E-14 | 73.5753 | cl26397 | NB-ARC superfamily | - |
| Transcript_14099 | specific | 227223 | 400 | 752 | 2.44E-07 | 54.2038 | COG4886 | LRR | - |
| Transcript_14099 | non-specific | 227223 | 592 | 753 | 2.62E-07 | 54.2038 | COG4886 | LRR | NC |
| Transcript_14099 | non-specific | 227223 | 591 | 753 | 1.35E-05 | 48.811 | COG4886 | LRR | C |
| Transcript_14099 | non-specific | 227223 | 595 | 755 | 0.00938662 | 39.5662 | COG4886 | LRR | C |
| Transcript_14099 | superfamily | 307194 | 51 | 296 | 9.24E-25 | 105.547 | cl26397 | NB-ARC superfamily | - |
| Transcript_1452 | specific | 227223 | 387 | 613 | 2.94E-05 | 47.2702 | COG4886 | LRR | N |
| Transcript_1452 | superfamily | 307194 | 63 | 322 | 4.05E-44 | 161.401 | cl26397 | NB-ARC superfamily | - |
| Transcript_14780 | specific | 227223 | 504 | 867 | 1.40E-08 | 58.441 | COG4886 | LRR | - |
| Transcript_14780 | non-specific | 227223 | 709 | 975 | 1.55E-08 | 58.0558 | COG4886 | LRR | C |
| Transcript_14780 | non-specific | 227223 | 759 | 1013 | 2.34E-08 | 57.6706 | COG4886 | LRR | C |
| Transcript_14780 | non-specific | 227223 | 761 | 1003 | 7.23E-08 | 56.1298 | COG4886 | LRR | C |
| Transcript_14780 | superfamily | 307194 | 4 | 325 | 1.88E-09 | 60.0933 | cl26397 | NB-ARC superfamily | - |
| Transcript_14916 | specific | 227223 | 563 | 699 | 0.00107338 | 42.6478 | COG4886 | LRR | NC |
| Transcript_14916 | non-specific | 227223 | 519 | 743 | 0.00354126 | 40.7218 | COG4886 | LRR | N |
| Transcript_14916 | superfamily | 307194 | 122 | 371 | 1.17E-24 | 105.162 | cl26397 | NB-ARC superfamily | - |
| Transcript_15017 | specific | 227223 | 903 | 1056 | 0.000568122 | 43.4182 | COG4886 | LRR | N |
| Transcript_15017 | non-specific | 227223 | 907 | 1125 | 0.00164612 | 41.8774 | COG4886 | LRR | N |
| Transcript_15017 | superfamily | 307194 | 490 | 753 | 1.85E-33 | 130.97 | cl26397 | NB-ARC superfamily | - |
| Transcript_15021 | specific | 227223 | 958 | 1035 | 0.00397825 | 40.7218 | COG4886 | LRR | NC |
| Transcript_15021 | superfamily | 307194 | 610 | 843 | 1.27E-38 | 145.608 | cl26397 | NB-ARC superfamily | - |
| Transcript_15023 | specific | 227223 | 727 | 969 | 4.26E-06 | 49.9666 | COG4886 | LRR | N |
| Transcript_15023 | superfamily | 307194 | 460 | 539 | 1.29E-09 | 60.4785 | cl26397 | NB-ARC superfamily | NC |
| Transcript_15026 | specific | 227223 | 1386 | 1646 | 0.00350915 | 41.4922 | COG4886 | LRR | N |
| Transcript_15026 | specific | 227223 | 883 | 1119 | 0.00831351 | 40.3366 | COG4886 | LRR | N |
| Transcript_15026 | superfamily | 307194 | 468 | 748 | 5.56E-34 | 132.896 | cl26397 | NB-ARC superfamily | - |
| Transcript_15026 | superfamily | 307194 | 1109 | 1230 | 6.52E-07 | 52.7745 | cl26397 | NB-ARC superfamily | N |
| Transcript_1506 | specific | 227223 | 223 | 501 | 1.93E-11 | 66.145 | COG4886 | LRR | - |
| Transcript_1506 | superfamily | 307194 | 558 | 709 | 2.87E-07 | 52.3893 | cl26397 | NB-ARC superfamily | C |
| Transcript_15242 | specific | 227223 | 1475 | 1659 | 5.27E-10 | 63.4486 | COG4886 | LRR | NC |
| Transcript_15242 | specific | 227223 | 1428 | 1583 | 0.00864702 | 40.3366 | COG4886 | LRR | NC |
| Transcript_15242 | superfamily | 307194 | 1048 | 1316 | 4.75E-28 | 115.562 | cl26397 | NB-ARC superfamily | - |
| Transcript_15380 | specific | 227223 | 293 | 601 | 5.72E-11 | 64.6042 | COG4886 | LRR | - |
| Transcript_15380 | non-specific | 227223 | 330 | 556 | 8.51E-06 | 48.0406 | COG4886 | LRR | N |
| Transcript_15380 | non-specific | 227223 | 247 | 403 | 0.000540705 | 42.6478 | COG4886 | LRR | NC |
| Transcript_15380 | superfamily | 307194 | 1 | 96 | 2.64E-06 | 49.3077 | cl26397 | NB-ARC superfamily | N |
| Transcript_15595 | specific | 227223 | 534 | 742 | 3.39E-05 | 46.885 | COG4886 | LRR | N |
| Transcript_15595 | non-specific | 227223 | 554 | 759 | 0.00197677 | 41.107 | COG4886 | LRR | C |
| Transcript_15595 | superfamily | 307194 | 140 | 412 | 1.08E-52 | 184.513 | cl26397 | NB-ARC superfamily | - |
| Transcript_16176 | specific | 227223 | 138 | 230 | 5.21E-05 | 44.959 | COG4886 | LRR | NC |
| Transcript_16176 | superfamily | 307194 | 1 | 61 | 1.31E-10 | 61.6341 | cl26397 | NB-ARC superfamily | N |
| Transcript_16194 | specific | 227223 | 462 | 581 | 6.29E-06 | 48.4258 | COG4886 | LRR | NC |
| Transcript_16194 | superfamily | 307194 | 31 | 326 | 1.41E-71 | 231.507 | cl26397 | NB-ARC superfamily | - |
| Transcript_16295 | specific | 227223 | 529 | 763 | 2.74E-10 | 63.4486 | COG4886 | LRR | N |
| Transcript_16295 | superfamily | 307194 | 121 | 361 | 1.21E-25 | 107.858 | cl26397 | NB-ARC superfamily | - |
| Transcript_16984 | specific | 227223 | 389 | 719 | 3.91E-06 | 49.9666 | COG4886 | LRR | C |
| Transcript_16984 | superfamily | 307194 | 5 | 250 | 1.03E-21 | 96.302 | cl26397 | NB-ARC superfamily | - |
| Transcript_18003 | specific | 227223 | 1487 | 1772 | 9.46E-09 | 60.367 | COG4886 | LRR | - |
| Transcript_18003 | specific | 227223 | 265 | 587 | 0.000108563 | 47.2702 | COG4886 | LRR | - |
| Transcript_18003 | specific | 227223 | 2877 | 2953 | 0.000348531 | 45.7294 | COG4886 | LRR | NC |
| Transcript_18003 | non-specific | 227223 | 2875 | 2951 | 0.000956726 | 44.1886 | COG4886 | LRR | NC |
| Transcript_18003 | superfamily | 307194 | 2664 | 2863 | 1.99E-41 | 155.238 | cl26397 | NB-ARC superfamily | C |
| Transcript_18003 | superfamily | 307194 | 131 | 240 | 1.15E-21 | 97.8428 | cl26397 | NB-ARC superfamily | NC |
| Transcript_18003 | superfamily | 307194 | 1431 | 1513 | 1.61E-10 | 64.7157 | cl26397 | NB-ARC superfamily | NC |
| Transcript_18003 | superfamily | 307194 | 916 | 983 | 2.11E-08 | 58.1673 | cl26397 | NB-ARC superfamily | NC |
| Transcript_18008 | specific | 227223 | 531 | 656 | 3.89E-05 | 46.885 | COG4886 | LRR | NC |
| Transcript_18008 | non-specific | 227223 | 585 | 815 | 0.000381124 | 43.8034 | COG4886 | LRR | NC |
| Transcript_18008 | non-specific | 227223 | 582 | 763 | 0.00148517 | 41.8774 | COG4886 | LRR | NC |
| Transcript_18008 | superfamily | 307194 | 174 | 466 | 1.92E-75 | 248.841 | cl26397 | NB-ARC superfamily | - |
| Transcript_18369 | specific | 227223 | 484 | 678 | 0.00535154 | 39.5662 | COG4886 | LRR | N |
| Transcript_18369 | superfamily | 307194 | 148 | 430 | 1.21E-70 | 231.892 | cl26397 | NB-ARC superfamily | - |
| Transcript_18500 | specific | 227223 | 439 | 519 | 0.00141795 | 42.2626 | COG4886 | LRR | NC |
| Transcript_18500 | superfamily | 307194 | 93 | 302 | 8.15E-37 | 140.6 | cl26397 | NB-ARC superfamily | N |
| Transcript_18647 | specific | 227223 | 602 | 776 | 1.00E-06 | 51.5074 | COG4886 | LRR | NC |
| Transcript_18647 | non-specific | 227223 | 572 | 766 | 4.12E-06 | 49.5814 | COG4886 | LRR | NC |
| Transcript_18647 | specific | 227223 | 39 | 169 | 0.000115395 | 44.959 | COG4886 | LRR | NC |
| Transcript_18647 | superfamily | 307194 | 207 | 389 | 2.80E-06 | 49.6929 | cl26397 | NB-ARC superfamily | C |
| Transcript_18752 | specific | 227223 | 416 | 632 | 4.30E-05 | 46.4998 | COG4886 | LRR | C |
| Transcript_18752 | superfamily | 307194 | 70 | 306 | 1.23E-18 | 87.0572 | cl26397 | NB-ARC superfamily | - |
| Transcript_19008 | specific | 227223 | 557 | 657 | 0.00093568 | 42.6478 | COG4886 | LRR | NC |
| Transcript_19008 | non-specific | 227223 | 524 | 639 | 0.00555183 | 40.3366 | COG4886 | LRR | NC |
| Transcript_19008 | superfamily | 307194 | 193 | 440 | 1.99E-54 | 191.446 | cl26397 | NB-ARC superfamily | - |
| Transcript_19106 | specific | 227223 | 160 | 319 | 6.51E-07 | 52.663 | COG4886 | LRR | NC |
| Transcript_19106 | non-specific | 227223 | 192 | 353 | 0.00448608 | 40.3366 | COG4886 | LRR | N |
| Transcript_19106 | superfamily | 307194 | 54 | 95 | 9.89E-08 | 54.3153 | cl26397 | NB-ARC superfamily | N |
| Transcript_19668 | specific | 227223 | 661 | 900 | 1.69E-06 | 51.8926 | COG4886 | LRR | C |
| Transcript_19668 | non-specific | 227223 | 685 | 899 | 4.27E-06 | 50.3518 | COG4886 | LRR | NC |
| Transcript_19668 | non-specific | 227223 | 650 | 878 | 5.50E-06 | 49.9666 | COG4886 | LRR | NC |
| Transcript_19668 | superfamily | 307194 | 223 | 471 | 3.72E-19 | 88.9832 | cl26397 | NB-ARC superfamily | - |
| Transcript_19706 | specific | 227223 | 145 | 389 | 5.05E-06 | 48.4258 | COG4886 | LRR | N |
| Transcript_19706 | non-specific | 227223 | 159 | 423 | 7.33E-05 | 44.959 | COG4886 | LRR | N |
| Transcript_19706 | superfamily | 307194 | 1 | 39 | 5.24E-09 | 57.0117 | cl26397 | NB-ARC superfamily | N |
| Transcript_20125 | specific | 227223 | 240 | 354 | 0.00720917 | 39.5662 | COG4886 | LRR | NC |
| Transcript_20125 | superfamily | 307194 | 1 | 129 | 3.21E-13 | 70.8789 | cl26397 | NB-ARC superfamily | N |
| Transcript_20235 | specific | 227223 | 597 | 819 | 0.000844917 | 42.2626 | COG4886 | LRR | NC |
| Transcript_20235 | superfamily | 307194 | 187 | 477 | 2.03E-48 | 173.342 | cl26397 | NB-ARC superfamily | - |
| Transcript_2047 | specific | 227223 | 196 | 327 | 5.84E-06 | 50.3518 | COG4886 | LRR | NC |
| Transcript_2047 | superfamily | 307194 | 62 | 103 | 3.51E-08 | 56.6265 | cl26397 | NB-ARC superfamily | N |
| Transcript_2048 | specific | 227223 | 500 | 698 | 8.41E-06 | 48.811 | COG4886 | LRR | N |
| Transcript_2048 | superfamily | 307194 | 164 | 416 | 2.40E-27 | 112.866 | cl26397 | NB-ARC superfamily | - |
| Transcript_20574 | specific | 227223 | 174 | 275 | 4.01E-08 | 53.4334 | COG4886 | LRR | NC |
| Transcript_20574 | non-specific | 227223 | 182 | 275 | 1.92E-07 | 51.5074 | COG4886 | LRR | NC |
| Transcript_20574 | non-specific | 227223 | 183 | 269 | 9.78E-07 | 49.1962 | COG4886 | LRR | NC |
| Transcript_20574 | non-specific | 227223 | 184 | 275 | 0.000150837 | 42.2626 | COG4886 | LRR | NC |
| Transcript_20574 | non-specific | 227223 | 175 | 257 | 0.000484364 | 40.7218 | COG4886 | LRR | NC |
| Transcript_20574 | non-specific | 227223 | 182 | 258 | 0.00180605 | 39.181 | COG4886 | LRR | NC |
| Transcript_20574 | superfamily | 307194 | 5 | 53 | 1.46E-10 | 60.0933 | cl26397 | NB-ARC superfamily | N |
| Transcript_20640 | specific | 227223 | 1381 | 1594 | 1.56E-06 | 52.2778 | COG4886 | LRR | NC |
| Transcript_20640 | specific | 227223 | 517 | 622 | 3.22E-05 | 48.0406 | COG4886 | LRR | NC |
| Transcript_20640 | non-specific | 227223 | 1359 | 1547 | 0.000487434 | 44.1886 | COG4886 | LRR | NC |
| Transcript_20640 | superfamily | 307194 | 167 | 431 | 2.22E-62 | 214.944 | cl26397 | NB-ARC superfamily | - |
| Transcript_20640 | superfamily | 307194 | 968 | 1248 | 1.96E-48 | 174.883 | cl26397 | NB-ARC superfamily | - |
| Transcript_20675 | specific | 227223 | 827 | 1054 | 2.17E-10 | 63.8338 | COG4886 | LRR | N |
| Transcript_20675 | non-specific | 227223 | 830 | 1023 | 4.89E-08 | 56.515 | COG4886 | LRR | N |
| Transcript_20675 | superfamily | 307194 | 447 | 703 | 1.60E-36 | 139.83 | cl26397 | NB-ARC superfamily | - |
| Transcript_21200 | specific | 227223 | 566 | 728 | 2.20E-05 | 47.6554 | COG4886 | LRR | NC |
| Transcript_21200 | superfamily | 307194 | 168 | 442 | 3.66E-55 | 192.987 | cl26397 | NB-ARC superfamily | - |
| Transcript_22202 | specific | 227223 | 554 | 714 | 8.53E-11 | 64.219 | COG4886 | LRR | NC |
| Transcript_22202 | non-specific | 227223 | 585 | 714 | 1.51E-07 | 54.2038 | COG4886 | LRR | NC |
| Transcript_22202 | non-specific | 227223 | 561 | 722 | 0.00969824 | 38.7958 | COG4886 | LRR | C |
| Transcript_22202 | superfamily | 307194 | 243 | 487 | 3.52E-34 | 132.126 | cl26397 | NB-ARC superfamily | - |
| Transcript_23078 | specific | 227223 | 518 | 683 | 8.06E-06 | 48.811 | COG4886 | LRR | NC |
| Transcript_23078 | non-specific | 227223 | 517 | 670 | 4.92E-05 | 46.4998 | COG4886 | LRR | N |
| Transcript_23078 | non-specific | 227223 | 358 | 582 | 0.000847508 | 42.6478 | COG4886 | LRR | N |
| Transcript_23078 | superfamily | 307194 | 2 | 168 | 3.83E-12 | 67.7973 | cl26397 | NB-ARC superfamily | N |
| Transcript_24284 | specific | 227223 | 570 | 715 | 2.50E-09 | 59.9818 | COG4886 | LRR | NC |
| Transcript_24284 | non-specific | 227223 | 532 | 867 | 2.38E-07 | 53.8186 | COG4886 | LRR | - |
| Transcript_24284 | non-specific | 227223 | 571 | 756 | 1.21E-06 | 51.5074 | COG4886 | LRR | N |
| Transcript_24284 | non-specific | 227223 | 509 | 657 | 0.000164877 | 44.959 | COG4886 | LRR | NC |
| Transcript_24284 | superfamily | 307194 | 180 | 472 | 3.72E-85 | 274.264 | cl26397 | NB-ARC superfamily | - |
| Transcript_24356 | specific | 227223 | 436 | 714 | 2.46E-09 | 59.5966 | COG4886 | LRR | - |
| Transcript_24356 | superfamily | 307194 | 93 | 347 | 9.59E-18 | 83.9756 | cl26397 | NB-ARC superfamily | - |
| Transcript_24434 | specific | 227223 | 594 | 813 | 4.41E-06 | 49.9666 | COG4886 | LRR | N |
| Transcript_24434 | superfamily | 307194 | 210 | 477 | 1.29E-38 | 145.608 | cl26397 | NB-ARC superfamily | - |
| Transcript_24435 | specific | 227223 | 378 | 470 | 0.00163601 | 41.107 | COG4886 | LRR | NC |
| Transcript_24435 | superfamily | 307194 | 2 | 249 | 2.29E-33 | 129.044 | cl26397 | NB-ARC superfamily | - |
| Transcript_24603 | specific | 227223 | 410 | 660 | 3.39E-05 | 47.2702 | COG4886 | LRR | NC |
| Transcript_24603 | superfamily | 307194 | 3 | 239 | 1.17E-19 | 90.1388 | cl26397 | NB-ARC superfamily | - |
| Transcript_290 | non-specific | 227223 | 839 | 1224 | 1.37E-11 | 68.0709 | COG4886 | LRR | - |
| Transcript_290 | superfamily | 307194 | 244 | 516 | 1.08E-23 | 102.465 | cl26397 | NB-ARC superfamily | - |
| Transcript_29916 | specific | 227223 | 352 | 429 | 0.00392588 | 39.9514 | COG4886 | LRR | NC |
| Transcript_29916 | superfamily | 307194 | 1 | 237 | 2.83E-41 | 151.771 | cl26397 | NB-ARC superfamily | - |
| Transcript_30968 | specific | 227223 | 514 | 693 | 0.000484211 | 44.1886 | COG4886 | LRR | NC |
| Transcript_30968 | superfamily | 307194 | 185 | 413 | 3.30E-38 | 144.837 | cl26397 | NB-ARC superfamily | - |
| Transcript_30973 | specific | 227223 | 566 | 708 | 4.16E-07 | 53.4334 | COG4886 | LRR | NC |
| Transcript_30973 | non-specific | 227223 | 519 | 715 | 8.47E-05 | 46.1146 | COG4886 | LRR | N |
| Transcript_30973 | superfamily | 307194 | 178 | 419 | 7.90E-38 | 143.296 | cl26397 | NB-ARC superfamily | - |
| Transcript_3440 | specific | 227223 | 360 | 654 | 6.64E-13 | 71.9229 | COG4886 | LRR | C |
| Transcript_3440 | non-specific | 227223 | 351 | 688 | 2.02E-12 | 70.3821 | COG4886 | LRR | - |
| Transcript_3440 | non-specific | 227223 | 323 | 539 | 2.43E-09 | 60.7522 | COG4886 | LRR | N |
| Transcript_3440 | superfamily | 307194 | 4 | 156 | 9.90E-16 | 78.9681 | cl26397 | NB-ARC superfamily | N |
| Transcript_34531 | specific | 227223 | 229 | 470 | 5.63E-09 | 58.441 | COG4886 | LRR | C |
| Transcript_34531 | non-specific | 227223 | 345 | 675 | 0.0012345 | 41.4922 | COG4886 | LRR | N |
| Transcript_34531 | superfamily | 307194 | 151 | 218 | 5.87E-05 | 45.4557 | cl26397 | NB-ARC superfamily | N |
| Transcript_34662 | specific | 227223 | 793 | 1147 | 1.12E-11 | 68.0709 | COG4886 | LRR | - |
| Transcript_34662 | non-specific | 227223 | 713 | 961 | 1.39E-10 | 64.6042 | COG4886 | LRR | N |
| Transcript_34662 | non-specific | 227223 | 1014 | 1149 | 0.00438345 | 40.7218 | COG4886 | LRR | NC |
| Transcript_34662 | superfamily | 307194 | 363 | 599 | 5.37E-23 | 100.539 | cl26397 | NB-ARC superfamily | - |
| Transcript_36702 | specific | 227223 | 44 | 319 | 9.29E-07 | 50.737 | COG4886 | LRR | - |
| Transcript_36702 | non-specific | 227223 | 40 | 168 | 2.49E-06 | 49.5814 | COG4886 | LRR | NC |
| Transcript_36702 | non-specific | 227223 | 36 | 247 | 2.50E-05 | 46.1146 | COG4886 | LRR | N |
| Transcript_36702 | non-specific | 227223 | 114 | 335 | 8.16E-05 | 44.5738 | COG4886 | LRR | C |
| Transcript_36702 | superfamily | 307194 | 362 | 493 | 9.17E-09 | 56.2413 | cl26397 | NB-ARC superfamily | C |
| Transcript_36805 | specific | 227223 | 305 | 485 | 1.40E-07 | 53.8186 | COG4886 | LRR | NC |
| Transcript_36805 | non-specific | 227223 | 316 | 431 | 0.00434714 | 39.5662 | COG4886 | LRR | NC |
| Transcript_36805 | superfamily | 307194 | 5 | 230 | 4.71E-28 | 114.021 | cl26397 | NB-ARC superfamily | - |
| Transcript_36806 | specific | 227223 | 375 | 463 | 0.000151041 | 43.8034 | COG4886 | LRR | NC |
| Transcript_36806 | non-specific | 227223 | 387 | 464 | 0.000392403 | 42.2626 | COG4886 | LRR | NC |
| Transcript_36806 | non-specific | 227223 | 350 | 464 | 0.0047851 | 38.7958 | COG4886 | LRR | NC |
| Transcript_36806 | non-specific | 227223 | 349 | 465 | 0.00536047 | 38.7958 | COG4886 | LRR | NC |
| Transcript_36806 | superfamily | 307194 | 14 | 236 | 1.49E-30 | 119.414 | cl26397 | NB-ARC superfamily | - |
| Transcript_37676 | specific | 227223 | 363 | 496 | 0.00624857 | 39.181 | COG4886 | LRR | NC |
| Transcript_37676 | superfamily | 307194 | 137 | 207 | 0.00020274 | 43.5297 | cl26397 | NB-ARC superfamily | N |
| Transcript_38596 | specific | 227223 | 368 | 525 | 2.66E-06 | 49.9666 | COG4886 | LRR | NC |
| Transcript_38596 | non-specific | 227223 | 384 | 573 | 0.000139168 | 44.5738 | COG4886 | LRR | NC |
| Transcript_38596 | superfamily | 307194 | 30 | 287 | 4.94E-43 | 157.164 | cl26397 | NB-ARC superfamily | - |
| Transcript_4024 | specific | 227223 | 380 | 644 | 0.000392178 | 43.8034 | COG4886 | LRR | C |
| Transcript_4024 | non-specific | 227223 | 541 | 771 | 0.00132996 | 41.8774 | COG4886 | LRR | C |
| Transcript_4024 | superfamily | 307194 | 37 | 264 | 3.70E-15 | 77.0421 | cl26397 | NB-ARC superfamily | - |
| Transcript_4042 | specific | 227223 | 795 | 978 | 2.62E-06 | 51.1222 | COG4886 | LRR | NC |
| Transcript_4042 | superfamily | 307194 | 317 | 523 | 3.03E-20 | 92.0648 | cl26397 | NB-ARC superfamily | C |
| Transcript_42198 | specific | 227223 | 311 | 500 | 0.000117644 | 45.3442 | COG4886 | LRR | N |
| Transcript_42198 | non-specific | 227223 | 338 | 603 | 0.00417959 | 40.3366 | COG4886 | LRR | C |
| Transcript_42198 | superfamily | 307194 | 6 | 184 | 1.06E-14 | 75.5013 | cl26397 | NB-ARC superfamily | N |
| Transcript_42226 | specific | 227223 | 553 | 726 | 2.72E-09 | 59.9818 | COG4886 | LRR | C |
| Transcript_42226 | non-specific | 227223 | 617 | 727 | 8.10E-06 | 49.1962 | COG4886 | LRR | NC |
| Transcript_42226 | superfamily | 307194 | 208 | 490 | 6.00E-56 | 194.913 | cl26397 | NB-ARC superfamily | - |
| Transcript_4364 | specific | 227223 | 357 | 547 | 0.000168817 | 44.1886 | COG4886 | LRR | NC |
| Transcript_4364 | superfamily | 307194 | 171 | 216 | 9.07E-07 | 50.8485 | cl26397 | NB-ARC superfamily | N |
| Transcript_44416 | specific | 227223 | 452 | 651 | 1.64E-05 | 47.6554 | COG4886 | LRR | C |
| Transcript_44416 | non-specific | 227223 | 453 | 639 | 0.000139651 | 44.5738 | COG4886 | LRR | N |
| Transcript_44416 | superfamily | 307194 | 118 | 380 | 1.69E-36 | 138.674 | cl26397 | NB-ARC superfamily | - |
| Transcript_45904 | specific | 227223 | 229 | 358 | 0.000536474 | 41.4922 | COG4886 | LRR | NC |
| Transcript_45904 | non-specific | 227223 | 246 | 358 | 0.00788901 | 37.6402 | COG4886 | LRR | NC |
| Transcript_45904 | non-specific | 227223 | 237 | 357 | 0.00839023 | 37.6402 | COG4886 | LRR | NC |
| Transcript_45904 | superfamily | 307194 | 1 | 164 | 1.36E-23 | 98.6132 | cl26397 | NB-ARC superfamily | N |
| Transcript_46345 | specific | 227223 | 702 | 998 | 3.87E-16 | 81.9381 | COG4886 | LRR | - |
| Transcript_46345 | non-specific | 227223 | 689 | 854 | 6.47E-12 | 68.8413 | COG4886 | LRR | NC |
| Transcript_46345 | specific | 227223 | 954 | 1189 | 1.22E-09 | 61.9078 | COG4886 | LRR | C |
| Transcript_46345 | non-specific | 227223 | 954 | 1166 | 5.58E-08 | 56.515 | COG4886 | LRR | NC |
| Transcript_46345 | superfamily | 307194 | 231 | 475 | 7.20E-19 | 88.598 | cl26397 | NB-ARC superfamily | - |
| Transcript_47133 | specific | 227223 | 252 | 419 | 1.23E-09 | 59.5966 | COG4886 | LRR | NC |
| Transcript_47133 | superfamily | 307194 | 7 | 106 | 4.86E-08 | 53.9301 | cl26397 | NB-ARC superfamily | N |
| Transcript_48173 | specific | 227223 | 350 | 517 | 3.32E-10 | 62.6782 | COG4886 | LRR | NC |
| Transcript_48173 | non-specific | 227223 | 363 | 592 | 3.80E-07 | 53.0482 | COG4886 | LRR | N |
| Transcript_48173 | superfamily | 307194 | 3 | 131 | 7.05E-12 | 66.6417 | cl26397 | NB-ARC superfamily | N |
| Transcript_4905 | specific | 227223 | 532 | 696 | 7.78E-13 | 70.7673 | COG4886 | LRR | NC |
| Transcript_4905 | non-specific | 227223 | 546 | 785 | 4.76E-11 | 65.3746 | COG4886 | LRR | NC |
| Transcript_4905 | superfamily | 307194 | 221 | 465 | 3.90E-34 | 132.126 | cl26397 | NB-ARC superfamily | - |
| Transcript_4954 | specific | 227223 | 656 | 846 | 6.97E-08 | 55.7446 | COG4886 | LRR | NC |
| Transcript_4954 | superfamily | 307194 | 301 | 475 | 7.52E-13 | 70.1085 | cl26397 | NB-ARC superfamily | N |
| Transcript_51859 | specific | 227223 | 390 | 564 | 5.19E-08 | 56.1298 | COG4886 | LRR | NC |
| Transcript_51859 | non-specific | 227223 | 391 | 611 | 6.48E-07 | 52.663 | COG4886 | LRR | N |
| Transcript_51859 | superfamily | 307194 | 7 | 251 | 2.26E-27 | 112.866 | cl26397 | NB-ARC superfamily | - |
| Transcript_5285 | specific | 227223 | 568 | 771 | 0.00710652 | 40.7218 | COG4886 | LRR | NC |
| Transcript_5285 | superfamily | 307194 | 302 | 426 | 2.27E-12 | 69.3381 | cl26397 | NB-ARC superfamily | NC |
| Transcript_53337 | specific | 227223 | 574 | 681 | 2.09E-05 | 47.6554 | COG4886 | LRR | NC |
| Transcript_53337 | non-specific | 227223 | 570 | 704 | 0.000213697 | 44.5738 | COG4886 | LRR | NC |
| Transcript_53337 | non-specific | 227223 | 552 | 679 | 0.00247868 | 41.107 | COG4886 | LRR | NC |
| Transcript_53337 | superfamily | 307194 | 163 | 456 | 2.44E-57 | 199.15 | cl26397 | NB-ARC superfamily | - |
| Transcript_56186 | specific | 227223 | 553 | 685 | 4.95E-11 | 66.145 | COG4886 | LRR | NC |
| Transcript_56186 | non-specific | 227223 | 550 | 671 | 5.80E-07 | 53.0482 | COG4886 | LRR | NC |
| Transcript_56186 | non-specific | 227223 | 535 | 646 | 1.43E-05 | 48.811 | COG4886 | LRR | NC |
| Transcript_56186 | non-specific | 227223 | 547 | 681 | 0.00407184 | 40.7218 | COG4886 | LRR | NC |
| Transcript_56186 | superfamily | 307194 | 170 | 423 | 3.91E-67 | 228.04 | cl26397 | NB-ARC superfamily | - |
| Transcript_56980 | specific | 227223 | 345 | 665 | 5.78E-10 | 62.293 | COG4886 | LRR | - |
| Transcript_56980 | non-specific | 227223 | 292 | 475 | 3.18E-08 | 56.515 | COG4886 | LRR | NC |
| Transcript_56980 | superfamily | 307194 | 15 | 200 | 7.91E-15 | 75.8865 | cl26397 | NB-ARC superfamily | N |
| Transcript_5774 | specific | 227223 | 523 | 665 | 0.000426028 | 43.4182 | COG4886 | LRR | NC |
| Transcript_5774 | superfamily | 307194 | 167 | 426 | 8.12E-40 | 148.689 | cl26397 | NB-ARC superfamily | - |
| Transcript_58010 | specific | 227223 | 142 | 251 | 1.94E-06 | 48.0406 | COG4886 | LRR | NC |
| Transcript_58010 | non-specific | 227223 | 154 | 250 | 5.75E-05 | 43.4182 | COG4886 | LRR | NC |
| Transcript_58010 | non-specific | 227223 | 115 | 251 | 0.000176692 | 41.8774 | COG4886 | LRR | NC |
| Transcript_58010 | superfamily | 307194 | 11 | 62 | 5.72E-05 | 43.1445 | cl26397 | NB-ARC superfamily | N |
| Transcript_58991 | specific | 227223 | 385 | 558 | 0.0064053 | 39.181 | COG4886 | LRR | NC |
| Transcript_58991 | superfamily | 307194 | 177 | 226 | 4.37E-05 | 45.4557 | cl26397 | NB-ARC superfamily | N |
| Transcript_59256 | specific | 227223 | 248 | 404 | 1.09E-10 | 64.219 | COG4886 | LRR | NC |
| Transcript_59256 | non-specific | 227223 | 242 | 470 | 1.38E-07 | 54.2038 | COG4886 | LRR | N |
| Transcript_59256 | non-specific | 227223 | 245 | 391 | 1.69E-07 | 54.2038 | COG4886 | LRR | N |
| Transcript_59256 | superfamily | 307194 | 2 | 120 | 4.60E-25 | 105.547 | cl26397 | NB-ARC superfamily | N |
| Transcript_59439 | specific | 227223 | 530 | 671 | 0.00193977 | 41.107 | COG4886 | LRR | NC |
| Transcript_59439 | superfamily | 307194 | 171 | 453 | 3.30E-71 | 235.744 | cl26397 | NB-ARC superfamily | - |
| Transcript_62224 | specific | 227223 | 628 | 776 | 8.66E-08 | 54.9742 | COG4886 | LRR | NC |
| Transcript_62224 | superfamily | 307194 | 228 | 449 | 3.48E-16 | 79.3533 | cl26397 | NB-ARC superfamily | - |
| Transcript_64179 | specific | 227223 | 138 | 228 | 2.14E-06 | 48.4258 | COG4886 | LRR | NC |
| Transcript_64179 | non-specific | 227223 | 148 | 274 | 7.43E-05 | 43.4182 | COG4886 | LRR | NC |
| Transcript_64179 | superfamily | 307194 | 1 | 44 | 4.61E-05 | 43.9149 | cl26397 | NB-ARC superfamily | N |
| Transcript_64230 | specific | 227223 | 240 | 415 | 1.61E-08 | 55.7446 | COG4886 | LRR | NC |
| Transcript_64230 | non-specific | 227223 | 263 | 392 | 0.000163981 | 43.4182 | COG4886 | LRR | NC |
| Transcript_64230 | non-specific | 227223 | 207 | 360 | 0.000306019 | 42.6478 | COG4886 | LRR | NC |
| Transcript_64230 | superfamily | 307194 | 27 | 132 | 1.69E-06 | 48.9225 | cl26397 | NB-ARC superfamily | N |
| Transcript_6564 | specific | 227223 | 582 | 857 | 1.18E-05 | 49.5814 | COG4886 | LRR | N |
| Transcript_6564 | superfamily | 307194 | 223 | 464 | 8.04E-18 | 85.5164 | cl26397 | NB-ARC superfamily | - |
| Transcript_67251 | specific | 227223 | 281 | 369 | 3.59E-05 | 45.3442 | COG4886 | LRR | NC |
| Transcript_67251 | non-specific | 227223 | 281 | 367 | 0.000369327 | 41.8774 | COG4886 | LRR | NC |
| Transcript_67251 | superfamily | 307194 | 1 | 146 | 2.71E-23 | 97.8428 | cl26397 | NB-ARC superfamily | N |
| Transcript_69073 | specific | 227223 | 599 | 779 | 0.00146726 | 41.8774 | COG4886 | LRR | C |
| Transcript_69073 | non-specific | 227223 | 635 | 760 | 0.00611112 | 39.9514 | COG4886 | LRR | NC |
| Transcript_69073 | superfamily | 307194 | 206 | 454 | 1.77E-25 | 107.473 | cl26397 | NB-ARC superfamily | - |
| Transcript_69424 | specific | 227223 | 561 | 732 | 0.0073155 | 39.5662 | COG4886 | LRR | NC |
| Transcript_69424 | superfamily | 307194 | 174 | 438 | 3.77E-67 | 225.729 | cl26397 | NB-ARC superfamily | - |
| Transcript_71378 | specific | 227223 | 597 | 696 | 2.86E-07 | 53.8186 | COG4886 | LRR | NC |
| Transcript_71378 | non-specific | 227223 | 583 | 695 | 2.12E-06 | 51.1222 | COG4886 | LRR | NC |
| Transcript_71378 | non-specific | 227223 | 593 | 695 | 2.83E-06 | 50.3518 | COG4886 | LRR | NC |
| Transcript_71378 | superfamily | 307194 | 188 | 479 | 4.00E-60 | 206.854 | cl26397 | NB-ARC superfamily | - |
| Transcript_71613 | specific | 227223 | 587 | 695 | 7.27E-06 | 48.811 | COG4886 | LRR | NC |
| Transcript_71613 | non-specific | 227223 | 591 | 695 | 0.000519684 | 43.033 | COG4886 | LRR | NC |
| Transcript_71613 | superfamily | 307194 | 206 | 488 | 8.86E-53 | 184.898 | cl26397 | NB-ARC superfamily | - |
| Transcript_71635 | specific | 227223 | 167 | 414 | 1.29E-06 | 50.3518 | COG4886 | LRR | N |
| Transcript_71635 | non-specific | 227223 | 156 | 373 | 7.39E-05 | 44.959 | COG4886 | LRR | NC |
| Transcript_71635 | superfamily | 307194 | 6 | 60 | 7.94E-14 | 71.6493 | cl26397 | NB-ARC superfamily | N |
| Transcript_71700 | specific | 227223 | 439 | 721 | 8.29E-06 | 49.1962 | COG4886 | LRR | C |
| Transcript_71700 | non-specific | 227223 | 545 | 758 | 0.000361268 | 44.1886 | COG4886 | LRR | C |
| Transcript_71700 | superfamily | 307194 | 139 | 379 | 6.73E-15 | 76.2717 | cl26397 | NB-ARC superfamily | - |
| Transcript_72010 | specific | 227223 | 223 | 446 | 0.000834749 | 41.4922 | COG4886 | LRR | N |
| Transcript_72010 | non-specific | 227223 | 195 | 365 | 0.00134833 | 41.107 | COG4886 | LRR | NC |
| Transcript_72010 | superfamily | 307194 | 1 | 57 | 4.86E-15 | 75.5013 | cl26397 | NB-ARC superfamily | N |
| Transcript_7215 | specific | 227223 | 559 | 656 | 5.93E-06 | 50.3518 | COG4886 | LRR | NC |
| Transcript_7215 | superfamily | 307194 | 151 | 430 | 1.37E-63 | 218.025 | cl26397 | NB-ARC superfamily | - |
| Transcript_72492 | specific | 227223 | 160 | 415 | 2.65E-08 | 55.7446 | COG4886 | LRR | N |
| Transcript_72492 | non-specific | 227223 | 147 | 238 | 1.79E-05 | 46.885 | COG4886 | LRR | NC |
| Transcript_72492 | superfamily | 307194 | 2 | 46 | 1.98E-14 | 73.5753 | cl26397 | NB-ARC superfamily | N |
| Transcript_7419 | specific | 227223 | 776 | 932 | 0.00768148 | 39.9514 | COG4886 | LRR | NC |
| Transcript_7419 | superfamily | 307194 | 198 | 458 | 9.64E-45 | 163.712 | cl26397 | NB-ARC superfamily | - |
| Transcript_77254 | specific | 227223 | 599 | 732 | 5.65E-10 | 62.293 | COG4886 | LRR | NC |
| Transcript_77254 | non-specific | 227223 | 607 | 848 | 1.18E-05 | 48.811 | COG4886 | LRR | N |
| Transcript_77254 | non-specific | 227223 | 599 | 757 | 2.35E-05 | 47.6554 | COG4886 | LRR | C |
| Transcript_77254 | non-specific | 227223 | 599 | 686 | 7.78E-05 | 46.1146 | COG4886 | LRR | NC |
| Transcript_77254 | superfamily | 307194 | 227 | 506 | 2.06E-58 | 202.232 | cl26397 | NB-ARC superfamily | - |
| Transcript_77300 | specific | 227223 | 255 | 406 | 4.78E-08 | 55.7446 | COG4886 | LRR | NC |
| Transcript_77300 | non-specific | 227223 | 255 | 384 | 1.78E-07 | 54.2038 | COG4886 | LRR | NC |
| Transcript_77300 | non-specific | 227223 | 245 | 381 | 4.56E-05 | 46.4998 | COG4886 | LRR | N |
| Transcript_77300 | non-specific | 227223 | 255 | 489 | 0.000607299 | 42.6478 | COG4886 | LRR | NC |
| Transcript_77300 | superfamily | 307194 | 3 | 133 | 9.08E-28 | 113.636 | cl26397 | NB-ARC superfamily | N |
| Transcript_77302 | specific | 227223 | 570 | 716 | 3.09E-08 | 56.9002 | COG4886 | LRR | NC |
| Transcript_77302 | superfamily | 307194 | 204 | 458 | 2.08E-50 | 179.89 | cl26397 | NB-ARC superfamily | - |
| Transcript_77360 | specific | 227223 | 302 | 445 | 0.00662584 | 39.5662 | COG4886 | LRR | NC |
| Transcript_77360 | superfamily | 307194 | 12 | 185 | 1.73E-23 | 101.31 | cl26397 | NB-ARC superfamily | N |
| Transcript_790 | specific | 227223 | 491 | 818 | 7.06E-09 | 58.441 | COG4886 | LRR | - |
| Transcript_790 | superfamily | 307194 | 193 | 477 | 5.68E-96 | 301.999 | cl26397 | NB-ARC superfamily | - |
| Transcript_8252 | specific | 227223 | 404 | 613 | 4.20E-05 | 46.4998 | COG4886 | LRR | NC |
| Transcript_8252 | non-specific | 227223 | 415 | 697 | 0.00136051 | 41.4922 | COG4886 | LRR | C |
| Transcript_8252 | superfamily | 307194 | 8 | 251 | 2.26E-24 | 103.621 | cl26397 | NB-ARC superfamily | - |
| Transcript_8461 | specific | 227223 | 358 | 491 | 0.00428087 | 39.5662 | COG4886 | LRR | NC |
| Transcript_8461 | superfamily | 307194 | 162 | 203 | 0.00187511 | 40.4481 | cl26397 | NB-ARC superfamily | N |
| Transcript_9039 | specific | 227223 | 606 | 732 | 0.000786089 | 42.6478 | COG4886 | LRR | NC |
| Transcript_9039 | superfamily | 307194 | 173 | 443 | 1.25E-85 | 275.42 | cl26397 | NB-ARC superfamily | - |
| Transcript_9053 | specific | 227223 | 588 | 740 | 2.36E-09 | 60.367 | COG4886 | LRR | NC |
| Transcript_9053 | superfamily | 307194 | 193 | 442 | 8.54E-29 | 117.103 | cl26397 | NB-ARC superfamily | - |
| Transcript_9069 | specific | 227223 | 560 | 738 | 1.92E-07 | 54.2038 | COG4886 | LRR | N |
| Transcript_9069 | non-specific | 227223 | 580 | 679 | 1.93E-07 | 54.2038 | COG4886 | LRR | NC |
| Transcript_9069 | non-specific | 227223 | 566 | 718 | 3.91E-06 | 50.3518 | COG4886 | LRR | NC |
| Transcript_9069 | superfamily | 307194 | 165 | 462 | 2.09E-58 | 202.232 | cl26397 | NB-ARC superfamily | - |
| Transcript_9077 | specific | 227223 | 406 | 550 | 1.12E-10 | 63.8338 | COG4886 | LRR | NC |
| Transcript_9077 | superfamily | 307194 | 53 | 325 | 1.40E-41 | 152.541 | cl26397 | NB-ARC superfamily | - |
| Transcript_9252 | specific | 227223 | 392 | 565 | 0.0032074 | 39.9514 | COG4886 | LRR | NC |
| Transcript_9252 | superfamily | 307194 | 191 | 233 | 0.000156862 | 43.9149 | cl26397 | NB-ARC superfamily | N |
| Transcript_9895 | specific | 227223 | 573 | 683 | 8.49E-10 | 62.293 | COG4886 | LRR | NC |
| Transcript_9895 | non-specific | 227223 | 581 | 669 | 5.51E-05 | 46.885 | COG4886 | LRR | NC |
| Transcript_9895 | specific | 227223 | 1130 | 1426 | 0.000300463 | 44.5738 | COG4886 | LRR | - |
| Transcript_9895 | non-specific | 227223 | 564 | 682 | 0.000574766 | 43.8034 | COG4886 | LRR | NC |
| Transcript_9895 | non-specific | 227223 | 595 | 682 | 0.00320744 | 41.4922 | COG4886 | LRR | NC |
| Transcript_9895 | superfamily | 307194 | 179 | 458 | 2.42E-64 | 220.336 | cl26397 | NB-ARC superfamily | - |

Supplementary table 2 Primers used in the amplification and RT-qPCR analysis

| Primer name^z^ | sequences | Amplification length |
| --- | --- | --- |
| *aDFR-F* | ATTAATTAGGCGGTGAGTTTGACATGG | 1055 |
| *aDFR-R* | GCTAGCAAAATGGGATCGGAGTC |  |
| *aCHI-F* | CAAATGGCTCCACCAATCACC | 722 |
| *aCHI-R* | CGGTTTCATGCCTCAACTTCTG |  |
| *aANR-F* | AACCATGGCCACCCACC | 1040 |
| *aANR-R* | ACCACCAGTTCTGGTTCTAGTTCTG |  |
| *aCYP-F* | AATGATCCTTAATATTGAGTGGCCTTGG | 1704 |
| *aCYP-R* | ATCCATGATGAACACCAATCTCACC |  |
| *aCER1-F* | GAAAAATGGCAACCACTCCTGG | 1919 |
| *aCER1-R* | GCGCAACAGATGATTAATTAAGCAGC |  |
| *aGPAT-F* | ACATCACTTTTCATCTAGGCGTC | 1141 |
| *aGPAT-R* | CGAAAGCATGAGCGTCCCT |  |
| *aMYB44-F* | GGCTTAATCAAAGCTTGTGTATGCC | 855 |
| *aMYB44-R* | CGGTTATGGATTCTTCTGCAATTGA |  |
| *qDFR-F* | TGTTGGAGACTGGGTTTG | 152 |
| *qDFR-R* | TTAGGCGGTGAGTTTGAC |  |
| *qCHI-F* | CATTGACGATTGGCTTCTC | 185 |
| *qCHI-R* | CACGCTATCATCACTCACT |  |
| *qANR-F* | AAAGCCAAGACAGTCAAAC | 123 |
| *qANR-R* | GGTCAAGAACTCAACATCAG |  |
| *qCYP-F* | TCGCCTCTGCTTGGGAAA | 180 |
| *qCYP-R* | CTCTGGTGATGATGACGCCTAT |  |
| *qCER1-F* | GGTGGTGGATGGAAGTAG | 106 |
| *qCER1-R* | GGCGTAAGCAACCTTTGTGAG |  |
| *qGPAT-F* | TGGAGAGCGTAGGTTGTATC | 120 |
| *qGPAT-R* | CGGGAAATATAAGGAGAGGGTTA |  |
| *qMYB44-F* | CACTTGCCGAGAAGACAGA | 120 |
| *qMYB44-R* | TTCCTAACCTCCTTCCTAATCATC |  |
| *qACT-F* | AGACTACATACAACTCCATCAT | 200 |
| *qACR-R* | ACCAATCCAGACACTATACTT |  |

^z^ The primers with a name initiated with little ‘a’ were used for amplification of the full length of transcript, while the primers with a name began with letter ‘q’ were used for quantitative PCR.
